# Phenotypic Characterization of Human Intermediate Monocytes

**DOI:** 10.3389/fimmu.2013.00339

**Published:** 2013-10-22

**Authors:** Daniëlle Hijdra, Adriane D. M. Vorselaars, Jan C. Grutters, Anke M. E. Claessen, Ger T. Rijkers

**Affiliations:** ^1^Department of Medical Microbiology and Immunology, St. Antonius Hospital, Nieuwegein, Netherlands; ^2^Department of Pulmonology, Centre for Interstitial Lung Diseases, St. Antonius Hospital, Nieuwegein, Netherlands; ^3^Division Heart and Lungs, University Medical Center Utrecht, Utrecht, Netherlands; ^4^Department of Sciences, University College Roosevelt, Middelburg, Netherlands

**Keywords:** monocyte subsets, classical monocytes, intermediate monocytes, non-classical monocytes, TNF receptors, TNFR1, TNFR2, sarcoidosis

A full understanding of the immune system requires full characterization of the cells, and its subsets, which play a role in the process of host defense. The expression pattern of cell surface molecules serves as a major criterion for assigning cells into discrete subsets. The availability of monoclonal reagents, specific fluorescent probes and compatible instrumentation over the past years has led to identification and characterization of a great number of leukocyte subsets, including monocyte subsets, recently reviewed by Ziegler-Heitbrock and Hofer (1).

When using flow cytometry, human blood monocytes are characterized by forward and side scatter as well as the expression of the LPS coreceptor CD14 and the Fcgamma receptor III CD16 (2). Based on the relative expression of CD14 and CD16, monocytes used to be subdivided in classical monocytes and CD16 expressing monocytes, of which the latter population is expanded in various diseases (3) [reviewed by Ref. (4)]. In 2008, a group of experts proposed (a nomenclature for) three subsets of blood monocytes: classical (CD14++CD16−), intermediate (CD14++CD16+), and non-classical (CD14+CD16++) (5). Since then, additional evidence from gene expression profiling and cell surface molecules underscored the existence of three monocyte subsets (6, 7).

Recently, Ziegler-Heitbrock and Hofer (1) presented an elegant review where they emphasized the importance of a refined analysis of the three different monocyte subsets. Intermediate monocytes are easily overlooked because of their small numbers and (inherent) transitional nature. It is still unknown if intermediate monocytes have a discrete, biologically meaningful role or whether they are the unavoidable intermediates in an otherwise continuous differentiation from classical into non-classical monocytes. Although the function of intermediate monocytes is not known, they could be important to tailor immune response in different pathologies, because intermediate monocytes have been found increased in certain conditions: in rheumatoid arthritis (8) and patients with severe asthma (9), intermediate monocytes are responsible for the increase in CD16 expressing monocytes. Recently, we have published that intermediate monocytes in blood are also increased in sarcoidosis (3.08 ± 0.25 versus 1.98 ± 0.22% in healthy controls; *P* = 0.017) (10).

Ziegler-Heitbrock and Hofer (1) correctly conclude that specific markers for the intermediate monocytes are required in order to unequivocally identify and enumerate this subset. In fact, data from our laboratory indicate that relative expression of TNF receptors and CC chemokine receptors can be used to further characterize intermediate monocytes. TNF is a major cytokine regulating the activity of monocytes and chemokines are essential for the selective recruitment of different leukocyte subsets to inflammatory sites. Therefore an extended phenotypic characterization of monocyte subsets, which includes expression of TNFR1, TNFR2, CCR2, and CCR5, can contribute to a more robust identification of intermediate monocytes.

**Figure 1:**
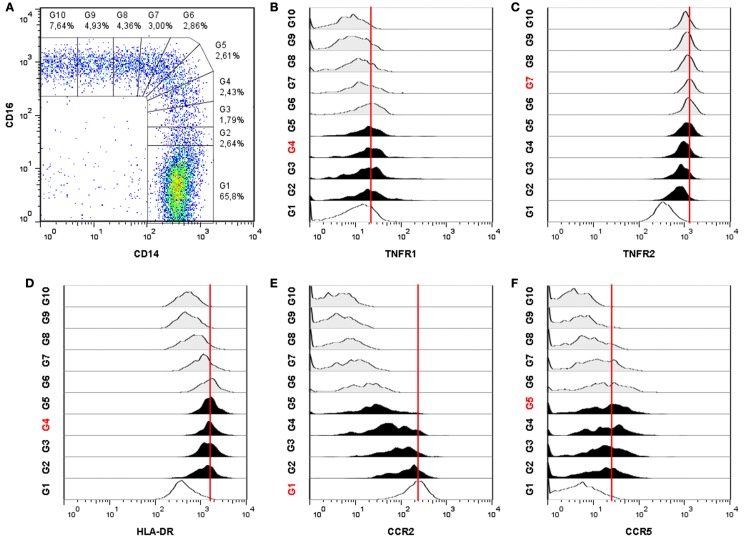
**Differential expression of TNFR1, TNFR2, HLA-DR, CCR2, and CCR5 on monocyte subsets of a sarcoidosis patient**. **(A)** Monocytes are subdivided into 10 gates based on an increasing CD16 expression and a decreasing CD14 expression. Monocyte subset tracking stages of TNFR1 **(B)**, TNFR2 **(C)**, HLA-DR, **(D)**, CCR2 **(E)**, and CCR5 **(F)**. The red gate number and the red line indicate the highest mean fluorescence intensity (MFI). Classical monocytes are identified by gate G1 (open histogram), intermediate monocytes by gates G2–G5 (filled histograms) and non-classical monocytes by gates G6–G10 (shaded histograms). The dotplot and all 5 histograms are representative for 52 individuals (sarcoidosis patients *n* = 39 and healthy controls *n* = 13).

In our experiments, we investigated the expression of TNFR1, TNFR2, HLA-DR, CCR2, and CCR5 on monocyte subsets using similar flow cytometric methods as proposed by Ziegler-Heitbrock and Hofer (1). In a four color set-up, cells were stained with anti-HLA-DR, CD14, CD16, and either TNFR1, TNFR2, CCR2, or CCR5. After staining, flow cytometry data were acquired on a FACSCalibur (BD Biosciences) and analyzed using FlowJo software (Tree Star, Ashland, OR, USA). Monocytes were gated based on forward × side scatter and side scatter × HLA-DR^+^ expression. Subsequently, monocytes were subdivided in 10 populations based on a gradual increasing CD16 and a decreasing CD14 expression pattern (Figure 1A). The multistep gating of intermediate and non-classical monocytes was used in order to obtain a more detailed picture of how the expression of TNFRs and CCRs will develop.

All blood monocytes, from normal healthy donors as well as from patients with sarcoidosis, expressed TNFR1. Highest expression of TNFR1 was found on intermediates (*P < *0.01; Figure 1B). All monocytes also expressed TNFR2, but the three subsets showed major differences in TNFR2 expression (*P < *0.0001; Figure 1C). Non-classical monocytes expressed the highest levels of TNFR2. Intermediates expressed less TNFR2 than non-classicals, but more than classicals. All monocytes expressed HLA-DR, but intermediate monocytes showed clearly a higher expression than classical and non-classical (*P < *0.0001; Figure 1D). Classical monocytes showed a high expression of CCR2, whereas non-classicals showed very low expression (Figure 1E). Intermediates did express CCR2, but in a lower level than classical. Intermediate monocytes also expressed the highest levels of CCR5 (Figure 1F). These data therefore indicate that intermediate monocytes differ from other monocyte subsets in expression levels of TNFR1, HLA-DR, CCR2, and CCR5.

The major challenge in understanding the full differentiation pathways of monocytes is that this pathway neither starts in the blood nor do the cells reach their final differentiated state in blood. The current available data indicate the presence of discrete subsets of monocytes in blood. The selective expression pattern of TNFR1, TNFR2, HLA-DR, CCR2, and CCR5 on intermediate monocytes would make this subset especially sensitive to cytokine and chemokine signals. The biological significance of these differences will need to be established in functional studies. Those can be either performed *in vitro* and/or by close monitoring of monocyte subsets in patients on anti-TNF medication.
